# Selective Partial Hydrolysis of 2-isopropyl-2-oxazoline Copolymers towards Decreasing the Ability to Crystallize

**DOI:** 10.3390/ma13153403

**Published:** 2020-08-01

**Authors:** Natalia Oleszko-Torbus, Barbara Mendrek, Agnieszka Kowalczuk, Alicja Utrata-Wesołek, Andrzej Dworak, Wojciech Wałach

**Affiliations:** Centre of Polymer and Carbon Materials, Polish Academy of Sciences, 34 M. Curie-Skłodowskiej St., 41-819 Zabrze, Poland; bmendrek@cmpw-pan.edu.pl (B.M.); akowalczuk@cmpw-pan.edu.pl (A.K.); autrata@cmpw-pan.edu.pl (A.U.-W.); adworak@cmpw-pan.edu.pl (A.D.); wwalach@cmpw-pan.edu.pl (W.W.)

**Keywords:** Poly(2-isopropyl-2-oxazoline), hydrolysis, crystallization, thermal properties

## Abstract

Poly(2-isopropyl-2-oxazoline) (PiPrOx) is readily prone to crystallization both in solid and from solutions. This feature is detrimental for certain applications. Here, we examine whether the presence of unsubstituted ethyleneimine (EI) units, a gradient distributed within a polymer chain composed of 2-isopropyl-2-oxazoline (iPrOx) and 2-methyl-2-oxazoline (MOx) units, decreases the ability to crystallize the copolymer and affects thermal properties compared to the homopolymer of iPrOx. We assumed that the separation of stiff iPrOx units by the more flexible EI will affect the spatial arrangements of the ordered chains, slightly plasticize and, as a result, decrease their ability to crystallize. The selective hydrolysis of gradient iPrOx and 2-methyl-2-oxazoline (MOx) copolymers, carried out under mild conditions, led to iPrOx/MOx/EI copolymers. To the best of our knowledge, the selective hydrolysis of these copolymers has never been carried out before. Their thermal properties and crystallization abilities, both in a solid state and from an aqueous solution, were analyzed. Based on the analysis of polymer charge and cytotoxicity studies, the potential use of the copolymers obtained was indicated in some biological systems.

## 1. Introduction

Poly(2-isopropyl-2-oxazoline) (PiPrOx) belongs to a large group of poly(2-substituted-2-oxazoline)s (POxs) obtained by cationic ring-opening polymerization, which are known from their peptidomimetic structure and bioinspired applications [1,2,3,4,5]. PiPrOx exhibits thermoresponsive behavior (Lower Critical Solution Temperature, LCST) near physiological temperature that can be modulated by appropriate functionalization [6,7] and thus is considered as a thermoresponsive pseudopeptide. Among POxs, although much research on excellent biocompatibility concerns mainly poly(2-methyl-2-oxazoline) (PMOx) and poly(2-ethyl-2-oxazoline) (PEtOx) [8,9,10,11,12], a lack of toxicity was also shown for the iPrOx-based (co)polymers towards different cell lines [12,13,14,15].

Despite these advantageous properties of PiPrOx, which make it attractive for biomedical applications, poly(2-isopropyl-2-oxazoline) is readily prone to crystallization in both solid and from solutions. The presence of a crystalline phase is detrimental for certain applications. The first systematic study on the crystallization of poly(2-oxazoline)s was reported by Litt et al. [16]. The crystallization mechanism of PiPrOx both in a solid state and from solutions was previously studied [17,18,19,20,21]. Generally, the ability to crystallize of PiPrOx involves the chemical structure of the polymer chain, which allows for easy packing and, as a consequence, ordering. The driving force for the crystallization of PiPrOx from solutions is an excess concentration (supersaturation) leading to the so-called polymer-rich phase state, maintained above equilibrium for a prolonged time (several hours). This issue was described in great detail for homo- and copolymers of iPrOx crystallized from aqueous solutions [22,23,24,25,26,27], but the crystallization of PiPrOx from organic [28] and water/organic mixtures [29] was also demonstrated. In the case of PiPrOx dissolved in aqueous solution or in a mixture of water with organic solvent, the local density of crystallizable macromolecules is increased when the liquid-liquid phase separation occurs above the LCST. When such a solution is incubated in a polymer-rich phase state for at least several hours, PiPrOx crystallites can be observed [23]. In the case of PiPrOx dissolved in organic solvents, as the supersaturation of the solution is not provided by the phase transition above the LCST (due to a lack of thermosensitivity in these solvents), the crystallization proceeds in more concentrated solutions for a more prolonged time (several weeks) [28]. Depending on the crystallization conditions, various morphologies of the isolated crystalline phase were observed: from fibrillar objects [28] to highly ordered microstructures known as “cotton balls” [23].

It was found that by the copolymerization of 2-isopropyl- with 2-n-propyl-2-oxazoline (nPrOx) (50 mol%), it was possible to obtain gradient copolymers with a significantly lower crystallization ability than PiPrOx, with only a slight change in LCST [26]. This property was explained by the higher elasticity of nPrOx units, which disturbed the spatial arrangements of the ordered iPrOx units.

In this work, we aimed to examine whether the presence of unsubstituted ethyleneimine (EI) units, a gradient distributed within the chain of iPrOx units, will decrease the ability to crystallize of copolymers and affect thermal properties compared to PiPrOx. It appears that the separation of stiff 2-isopropyl-2-oxazoline units by the more flexible EI will affect the spatial arrangements of the ordered chain, slightly plasticizing and decreasing the ability to crystallize, similar to the case of iPrOx/nPrOx copolymers.

To obtain ethyleneimine units in the poly(2-oxazoline) chain, hydrolysis is necessary. Acidic and basic hydrolysis conditions, leading to full or partial cleavage of the bond between backbone and substituent (side groups), were studied for many POxs [13,30,31,32,33,34,35,36,37,38].

In these studies, to obtain the copolymer of iPrOx with an EI unit gradient distributed within the chain, the selective hydrolysis of iPrOx/MOx gradient copolymers was carried out. The selective hydrolysis of iPrOx/MOx gradient copolymers has never been carried out previously. We assumed that this reaction could be performed under mild, basic conditions and conventional heating, yielding the easy cleavage of 2-methyl-2-oxazoline substituents while preserving iPrOx side groups. The thermal properties of iPrOx/MOx (before hydrolysis) and iPrOx/MOx/EI (after hydrolysis) copolymers and their crystallization ability, both in a solid state and from aqueous solutions, were analyzed. Here, we also checked whether the obtained iPrOx/MOx/EI gradient copolymers could potentially be applied in biological systems.

## 2. Materials and Methods

Materials: Isobutyronitrile (99.6%, Aldrich, Steinheim, Germany), 2-aminoethanol (99%, Aldrich, Steinheim, Germany), cadmium acetate (>98%, Fluka, Steinheim, Germany), methyl 4-nitrobenzenesulfonate (99%, Aldrich, Steinheim, Germany), KOH (85%, POCH, Gliwice, Poland), and NaOH (czda-basic 98.8%, POCH, Gliwice, Poland) were used as received. 2-Isopropyl-2-oxazoline was synthesized according to Witte and Seeliger [39]. Briefly, an equimolar amount of isobutyronitrile was added to 2-aminoethanol, and the mixture was heated under reflux in the presence of cadmium acetate. The amounts of reagents were checked by gas chromatography, and after full conversion, the mixture was distilled under a dry argon atmosphere. Raw 2-isopropyl-2-oxazoline (iPrOx) and 2-methyl-2-oxazoline (MOx, 98%, Aldrich, Steinheim, Germany) were dried over KOH, distilled, dried over CaH_2_ and distilled again. Acetonitrile (for HPLC, POCH, Gliwice, Poland) was dried over CaH_2_ and distilled under a dry argon atmosphere. Fibrosarcoma HT1080 cells (ATCC, Manassas, VA, USA) were cultured in Eagle’s Minimum Essential Medium (EMEM, ATCC, Manassas, VA, USA) with 10% Fetal Bovine Serum (ATCC, Manassas, VA, USA), penicillin (10,000 U/mL), streptomycin (10 mg/mL) and amphotericin B (25 µg/mL, PAA Laboratories Inc, Toronto, ON, Canada). Alamar Blue (Biosource International Inc, Camarillo, CA, USA) was used as received.

Synthesis of copolymers: Gradient copolymers of iPrOx with MOx were obtained via cationic ring-opening polymerization initiated by methyl 4-nitrobenzenesulfonate, according to a procedure described previously [15]. The ratio of the initial monomer concentration to the initiator concentration was chosen to be 100:1 for all syntheses. Three compositions of comonomers were applied with MOx contents equal to 10, 20, and 50 units. For all syntheses, acetonitrile was used as the solvent. Polymerizations were carried out at 75 °C to full conversion of the monomers (checked by gas chromatography). Then, water was added, the mixture was kept for 10 min at room temperature under stirring, the excess acetonitrile was evaporated, and the obtained polymer was dried by lyophilization. Copolymers with narrow molar mass dispersity (*Ð*) were obtained, and good agreement of molar masses (M_n_)with the theoretical values was achieved (determined by Gel permeation chromatography with multiangle laser light scattering detector, GPC MALLS). The degree of polymerization (DP) of copolymers was calculated on the basis of monomer feed and nuclear magnetic resonance (NMR) data. The reactivity ratios calculated basing on the monomer consumption rate [40] clearly indicated the gradient structure of iPrOx/MOx copolymers. The symbols, amounts of comonomers, molar masses and dispersity of the obtained copolymers are provided in Table 1.

Hydrolysis of copolymers: The iPrOx/MOx copolymers were dissolved in water at a concentration of 10 g/L. A 10-fold molar excess of NaOH or KOH in relation to the MOx unit was applied for each reaction. The hydrolysis reactions were performed via conventional heating. The hydrolysis of P(iPrOx-MOx)1 and P(iPrOx-MOx)2 was carried out for 20 h with the use of KOH at a temperature of 100 °C under reflux. In the case of P(iPrOx-MOx)3, hydrolysis proceeded for 14 days with the use of NaOH at 65 °C. The progress of hydrolysis was evaluated by ^1^H NMR on the basis of decreasing intensity of peaks assigned to the methyl protons of the MOx side groups. After hydrolysis, the copolymers were desalinated by ion exchangers (Merck) and then dried by lyophilization.

Measurements: The molar mass and molar mass dispersity (*Ð*) of the copolymers were determined using a GPC-MALLS system with a multiangle laser light scattering detector (DAWN EOS, Wyatt Technologies, Santa Barbara, CA, USA, λ = 658 nm) and a refractive index detector (Δn-1000 RI WGE DR Bures, Dallgow, Germany, λ = 620 nm). Measurements were carried out in *N,N*-dimethylformamide (DMF) (POCH, Gliwice, Poland) (with 5 mmol/L of LiBr; flow rate of 1 mL/min) using PSS 100 Å, 1000 Å and 3000 Å GRAM columns. The refractive index increment (*dn/dc*) was measured in DMF independently using a SEC-3010 detector (WGE DR Bures, Dallgow, Germany) (λ = 620 nm).

Differential scanning calorimetry (DSC) measurements were carried out using a TA-DSC Q2000 apparatus (TA Instruments, New Castle, DE, USA) under a nitrogen atmosphere with a flow rate of 50 mL/min. The measurements were taken in the range from 0 to 200 °C. The heating rate for the standard measurement was 10 °C/min and was equal to 2.5 °C/min for the so-called “slow-heating” measurement. To determine the glass transition temperature (T_g_) of the copolymer, after the first run, the sample was quenched with liquid nitrogen, and a second heating run was performed again from 0 to 200 °C. The enthalpy of melting or crystallization (ΔH) was calculated as the area under the peak, limited by the baseline. The data were collected and then analyzed using Universal Analysis 2000 with Universal V4.5a software (TA Instruments, New Castle, DE, USA).

Turbidimetric measurements of the aqueous solution of copolymers were performed using a Specord 200 plus ultraviolet–visible (UV–Vis) spectrophotometer (Analytik Jena, Jena, Germany) equipped with a programmable thermocontroller. The transmittance of the polymer solutions was monitored at a wavelength λ = 550 nm as a function of temperature (heating and then cooling cycle) with constant stirring of the solution. Transmittance values were recorded every 1 °C after 60 s of temperature stabilization. The phase transition temperature (T_CP_) value was defined as the temperature at which the transmittance of the copolymer solutions reached 50% of its initial value. Copolymer solutions were passed through 0.2 μm filters before measurement.

The sample crystallinity was measured by a wide-angle X-ray diffractometer (WAXS) TUR-M62 (VEB TuR, Dresden, Germany) equipped with an HZG-3 goniometer using Cu Kα radiation. Vaseline was used as a background. Calculations of the intensities and positions of peaks were carried out using WAXSFIT software (WAXSFIT, Bielsko Biała, Poland). Preparation of samples for X-ray analysis: copolymers in a solid state were annealed at 150 °C for 1 h and then cooled to room temperature at a rate of 10 °C/min. Aqueous solutions of the copolymers (c = 5 g/L) were incubated for 12 h at 70 °C (in the polymer-rich phase state), frozen in liquid nitrogen and dried by lyophilization.

Scanning electron microscopy (SEM) analysis was performed using an ESEM Quanta 250 FEG (FEI Co., Hillsboro, OR, USA) microscope with an Everhart–Thornley detector (ETD) under high vacuum mode. Preparation of the samples for SEM: 10μL aliquots of copolymer water suspensions (c = 1 g/L) preheated for 12 h at 70 °C (in the polymer-rich phase state) were placed on a mica surface, frozen in liquid nitrogen and lyophilized.

The composition of the copolymers and the progress of the hydrolysis were analyzed by ^1^H NMR. The spectra were recorded using a Bruker Ultrashield (Bruker, Billerica, MA, USA) spectrometer operating at 600MHz in D_2_O or CDCl_3_ as the solvent.

Zeta potential measurements for P(iPrOx-MOx-EI)1 were performed on a Zetasizer Nano ZS 90 (Malvern Instruments, Malvern, UK) in a disposable folded capillary cell. The aqueous solution (c =5 g/L) was passed through a 0.2 μm filter before use. Measurements were performed in triplicate at 25 °C, and then the temperature was raised to 65 °C. After 30 min of stabilization, measurements were performed in triplicate again. The zeta potential (*ζ*) was calculated from the electrophoretic mobility, *u*, employing the Helmholtz–Smoluchowski equation (u = εζ/η, where *ε* is the dielectric constant of the solvent and *η* is the viscosity of the solvent).

Cytotoxicity test: Fibrosarcoma HT1080 cells were seeded on 96-well plates at a density of 3300/well in 4 replicates. P(iPrOx-MOx-EI)1 was added to the culture medium to obtain solutions at concentrations of 0.001, 0.01, 0.1, 1 and 10 mg/mL. After 4, 8, 24, 48 and 72 h of incubation, the medium was removed, cells were washed with phosphate-buffered saline (PBS), and medium with AlamarBlue (10%) was added. One hour after the addition of AlamarBlue, fluorescence was measured, and the number of cells was determined based on the difference in the absorption of wavelengths at 560 nm and 590 nm using a Victor X5 automatic fluorescence reader (Perkin Elmer, Waltham, MA, USA). A standard curve was prepared, which was used to determine the number of cells cultured on the tested material.

## 3. Results

It is known that a homopolymer of iPrOx exhibits a high ability to crystallize both in the solid state and from solutions. The presence of an insoluble crystalline phase may be unfavorable, for example, for applications where the thermoresponsive behavior of the polymer is used and returning to transparent of solution is needed. In these studies, gradient copolymers of iPrOx with MOx with different comonomer ratios were obtained. Based on the literature studies [35,36,37], we assumed that their selective hydrolysis will lead to copolymers of iPrOx with ethyleneimine (EI) units of different stiffness and mobility compared to MOx. The aim of the work was to determine how the presence of relatively stiff (MOx) and elastic (EI) units influences the thermal properties of iPrOx copolymers and their ability to crystallize both in the solid state and from water.

### 3.1. Thermal and Crystalline Properties of 2-Isopropyl-2-Oxazoline/2-Methyl-2-Oxazoline (iPrOx/MOx) Copolymers

#### 3.1.1. Copolymers iPrOx/MOx in the Solid State

**Figure 1:**
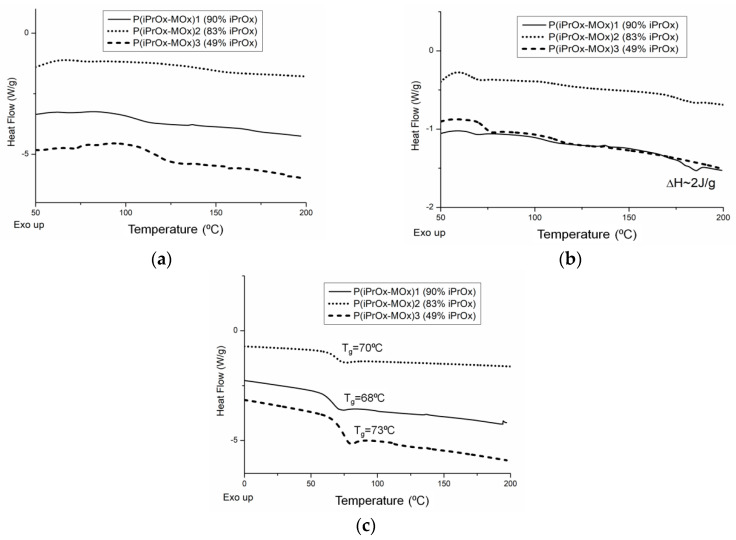
DSC traces for 2-isopropyl-2-oxazoline/2-methyl-2-oxazoline (iPrOx/MOx) copolymers at heating rates of (**a**) 10 °C/min and (**b**) 2.5 °C/min; (**c**) second DSC heating run (10 °C/min) after quenching of copolymers.

Gradient iPrOx copolymers with MOx (Table 1) were analyzed in terms of their thermal and crystalline properties both directly after synthesis (without “thermal history”) and after annealing at certain times and temperature. DSC traces for copolymers without thermal treatment are presented in Figure 1. It is worth noting that for all copolymers, neither exo- nor endothermic peaks, derived from crystallization and melting, respectively, could be seen during the standard DSC measurement conditions (heating rate of 10 °C/min, Figure 1a). This indicates that iPrOx copolymers with MOx exhibit a decreased ability to crystallize compared with the iPrOx homopolymer. However, during the “slow-heating” DSC measurement (heating rate of 2.5 °C/min, Figure 1b), a very small melting peak in the temperature range 170–180 °C was observed for the copolymer with the highest amount of iPrOx (P(iPrOx-MOx)1). This suggests that a small amount of the polymer fraction (not detected by X-ray diffraction) was ordered during the “slow heating” measurement and then melted. Increasing the amount of MOx in copolymers (relatively rigid monomer units compared to iPrOx) caused an anti-plasticizing effect, and a slight increase in the glass transition from 68 °C (10 mol% MOx) to 73 °C (51 mol% MOx) was observed (Figure 1c).

**Figure 2:**
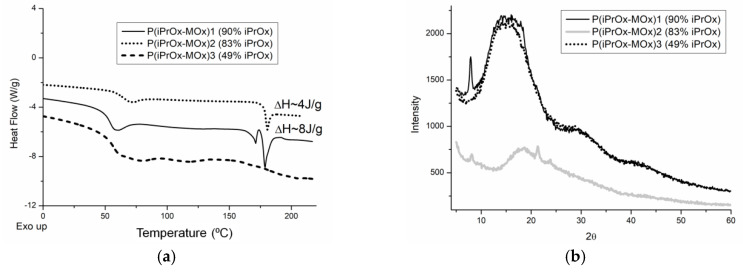
(**a**) DSC traces (heating rate of 10 °C/min) and (**b**) X-ray diffraction curves for iPrOx/MOx copolymers annealed at 150 °C for 1 h.

It is known that the amount of crystalline phase in many POxs can be increased by annealing for a prolonged time and at an elevated temperature [26,28]. To check how much the ability to crystallize iPrOx/MOx copolymers increases after the thermal treatment, the copolymers were annealed at 150 °C for 1 h and then cooled slowly to room temperature at a rate of 10 °C/min. After this procedure, endothermic peaks in the DSC curve, ascribed to melting, appeared in the temperature range of 170–180 °C for the copolymers with 90% and 83 mol% of iPrOx (Figure 2a). At the same time, for these copolymers, small diffraction peaks in the XRD curve could be seen at 2θ = 7.9–8.0° (*d* = 11.0–11.2 Å) (Figure 2b). A peak at a similar value of 2θ was also observed in the diffractograms of PiPrOx [28,29]. However, while the degree of crystallinity for PiPrOx after similar thermal treatment was approximately 68% [28], the amount of crystalline phase for the iPrOx/MOx copolymers was very small and was not possible to determine.

#### 3.1.2. iPrOx/MOx Copolymers in Aqueous Solution

**Figure 3:**
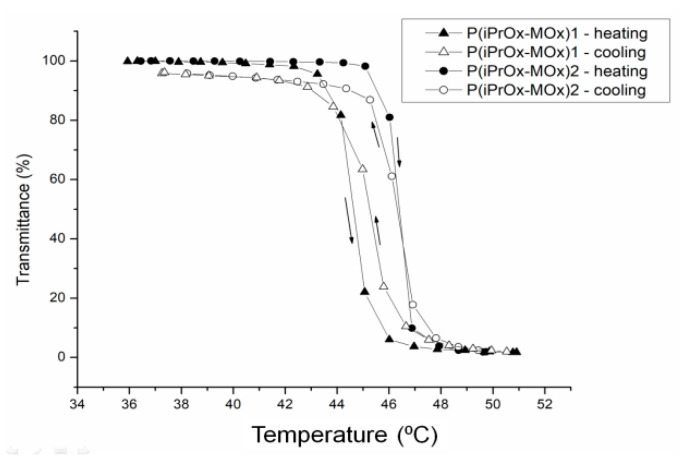
Transmittance-temperature dependence during heating (filled signs) and cooling (empty signs) of aqueous solutions of copolymers (c = 5 g/L).

The thermosensitivity of iPrOx/MOx copolymers was studied in water at different concentrations (Table 2). An increasing amount of MOx in the copolymer caused an increase in the cloud point temperature. The phase transition was completely reversible when the solution heated above T_CP_ was then cooled to room temperature, and practically no hysteresis of transition was observed (Figure 3).

**Figure 4:**
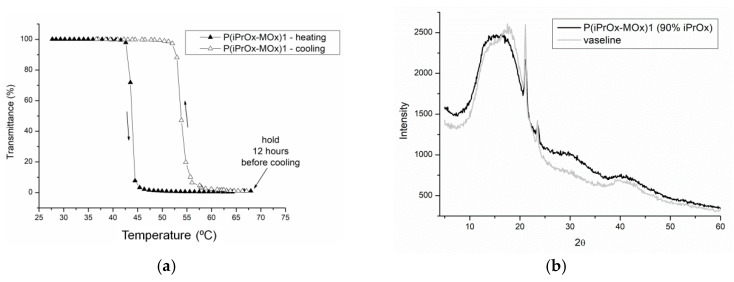
(**a**) Transmittance-temperature dependence during heating (filled triangles) and cooling (empty triangles) of 5 g/L aqueous solutions of P(iPrOx-MOx)1 (solution kept for 12 h at 70 °C before cooling); (**b**) X-ray diffraction curves of P(iPrOx-MOx)1 incubated for 12 h at 70 °C.

As shown in many studies, the prolonged incubation of the solutions of some POxs based on 2-isopropyl-2-oxazoline above T_CP_ leads to crystallization [22,23,24,25,26,27]. The liquid-liquid phase separation driven by the change in temperature above T_CP_ increases the local density of crystallizable macromolecules, thus leading to supersaturation and crystallization. This was observed for iPrOx homopolymers with different chain ends [22,23,24,25], gradient iPrOx copolymers with n-propyl-2-oxazoline at an amount of nPrOx equal to or less than 15 mol% [26], and for block iPrOx copolymers with MOx with a degree of polymerization of 50 for each block [27]. In this work, aqueous solutions of gradient iPrOx copolymers with MOx (P(iPrOx-MOx)1 and P(iPrOx-MOx)2) were incubated for 12 h at 70 °C, a temperature above T_CP_. Although the solutions were incubated in the polymer-rich phase state for a prolonged time, no precipitation was observed when the temperature was lowered to room temperature. A full return to transparency of the solutions was observed, which was confirmed by turbidity measurements (an exemplary transmittance-temperature curve for P(iPrOx-MOx)1 is presented in Figure 4a). Macroscopically, this observation indicated a lack of crystallization in the solution, confirming the conclusion drawn from the WAXS curves, where no diffraction peaks were seen (an exemplary diffraction curve for P(iPrOx-MOx)1 is presented in Figure 4b). However, a pronounced hysteresis of the phase transition in water, at approximately 10 °C, was found. Additionally, it should be emphasized that the observed hysteresis is “reversed”: the transition during heating occurs at a lower temperature than the transition during cooling of the system. Such behavior was already observed for some poly(2-oxazoline)s [40,41] and was explained by the organization of the polymer chains into specific structures. Most likely, at certain concentrations, agglomerates of micelle-like structures are formed, but they are not stable during cooling; thus, their disintegration occurred at higher temperatures than their formation during heating [40].

**Figure 5:**
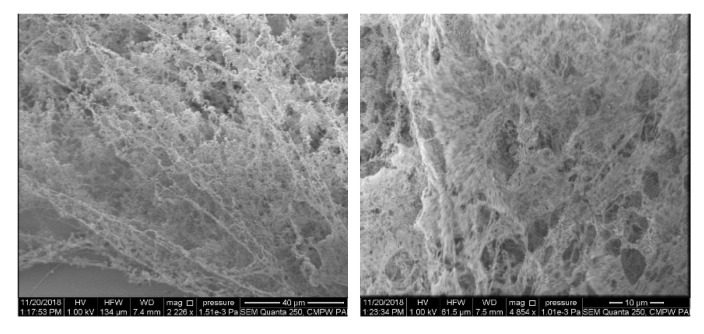
Scanning electron microscopy (SEM) micrographs of P(iPrOx-MOx)1 annealed in water for 12 h at the polymer-rich phase state.

Microscopic observations of iPrOx/MOx copolymers incubated in water at the polymer rich-phase state indicated the lack of ordered, crystalline structures. Exemplary SEM micrographs for P(iPrOx-MOx)1) incubated in water for 12 h at 70 °C are presented in Figure 5. Neither regular fibrillar objects nor so-called “cotton balls”, as in the case of crystalline PiPrOx [23], were observed.

Briefly, iPrOx copolymers with MOx revealed a significantly decreased ability to crystallize in the solid state compared to the iPrOx homopolymer. Crystallization of thermoresponsive iPrOx/MOx copolymers in water was also not observed. However, after the prolonged heating of the aqueous solution of thermoresponsive iPrOx/MOx copolymers, a pronounced hysteresis of the phase transition was found. This may not be desirable for applications where the T_CP_ of a polymer solution is needed at a certain temperature. It seemed interesting to determine how the presence of flexible non-substituted ethyleneimine (EI) units within the copolymer chain affects the crystallization ability in the solid state and in aqueous solution in comparison to iPrOx/MOx copolymers. It was also interesting to check whether the copolymers with EI maintained thermosensitivity in aqueous solution and wide hysteresis of the phase transition after prolonged heating.

### 3.2. Selective Hydrolysis of iPrOx/MOx Copolymers in Basic Aqueous Solution

The selective hydrolysis of POxs leads to the cleavage of strictly defined substituents under appropriate conditions. Research on the selective hydrolysis of POxs mainly concerns the gradient and diblock copolymers of 2-methyl-2-oxazoline with 2-phenyl-2-oxazoline (PhOx) [35,36,37], but studies with the use of the block copolymers of 2-ethyl-2-oxazoline with 2-tert-butylbenzoyl-2-oxazoline or with 2-undecyl-2-oxazoline [38] are also known. For the gradient and diblock MOx/PhOx copolymers, it was shown that under acidic conditions and microwave irradiation, both 2-methyl- and 2-phenyl-2-oxazoline substituents were readily cleaved, while under basic conditions and conventional heating, PhOx side groups were almost not hydrolyzed, leading to higher selectivity [35]. On the other hand, for similar copolymers, selective hydrolysis was carried out under acidic conditions and microwave irradiation in an ethanol-water 80:20 (*v*/*v*) mixture [36]. A high level of selectivity was achieved, as 95% of the MOx substituents were hydrolyzed while preserving most of the PhOx side groups, with a hydrolysis degree of only 10%. However, a lower degree of selective hydrolysis was observed when the reaction was carried out under acidic conditions and via conventional heating [37].

**Figure 6:**
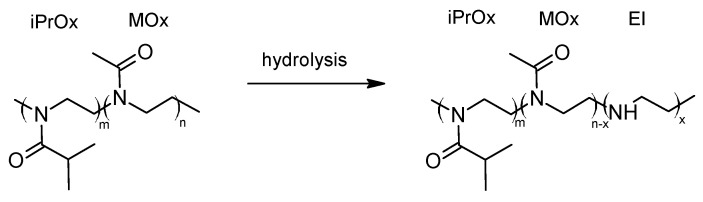
The selective alkaline reaction of hydrolysis.

Based on these results, we carried out basic hydrolysis of the iPrOx/MOx copolymers under conventional heating to provide selectivity for the cleaved groups. The hydrolysis conditions were chosen in such a way that the amide bond of the MOx substituent could be easily hydrolyzed, leading to ethyleneimine units while preserving the iPrOx groups (Figure 6).

Mild conditions. First, we used NaOH as the cleaving agent. At temperatures above T_CP_, the polymer undergoes a coil-to-globule transition, and it appears that easily accessible MOx substituents subjected to hydrolysis may become harder to cleave. For this reason, hydrolysis was initially carried out at temperatures below T_CP_ (42 °C for P(iPrOx-MOx)1 and 43 °C for P(iPrOx-MOx)2, c = 10 g/L). However, under these conditions, we did not observe the progress of hydrolysis, even after several days. In the case of P(iPrOx-MOx)3 (T_CP_ > 100 °C), hydrolysis with NaOH proceeded at 65 °C, and after 14 days, approximately 30% of the side groups of MOx were cleaved (based on ^1^H NMR, data not shown). After this time, the reaction stopped, and no further hydrolysis was observed. We concluded that an increase in temperature is necessary for efficient hydrolysis; however, we were still limited by the relatively low T_CP_ value in the case of P(iPrOx-MOx)1 and P(iPrOx-MOx)2. The addition of ethanol to the aqueous solution of copolymers led to a slight increase in T_CP_ (58 and 59 °C for P(iPrOx-MOx)1 and P(iPrOx-MOx)2, respectively, 30 v/v% of EtOH, c = 10 g/L), and thus, the temperature of hydrolysis could be raised. However, we still did not observe reaction progress for these copolymers after several days. Moreover, hydrolysis did not occur even after increasing the temperature far above the T_CP_ of copolymers, up to 80 °C. It seems that a relatively low amount of MOx side groups in the case of P(iPrOx-MOx)1 (10 mol%) and P(iPrOx-MOx)2 (17 mol%) is probably shielded by the isopropyl groups in the solution, and thus, at relatively mild conditions (NaOH), hydrolysis could not proceed.

**Figure 7:**
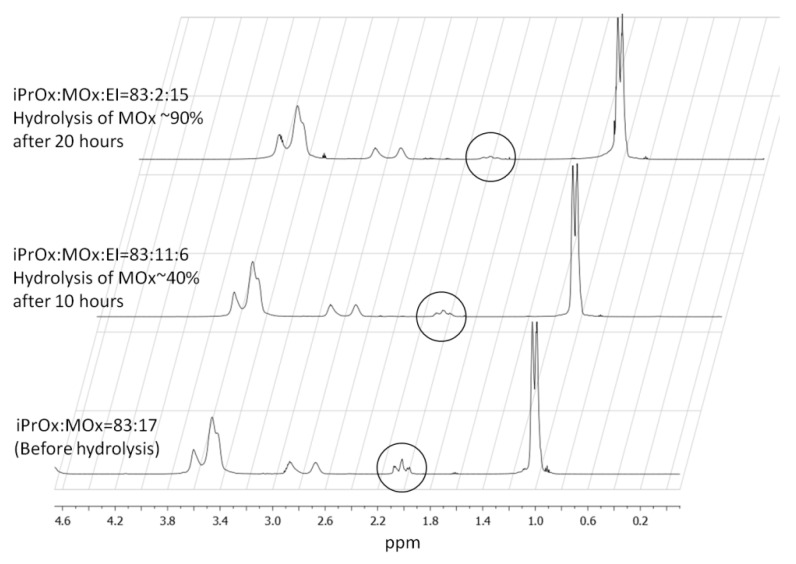
^1^H NMR spectra of P(iPrOx-MOx)2 during hydrolysis (CDCl_3_).

Stricter conditions. In the next step, a slightly stronger base, KOH, was applied. The temperature and time of hydrolysis were optimized considering the possible degradation of the polymer chains. First, the hydrolysis reaction was carried out below the T_CP_ of copolymers (42 and 43 °C for P(iPrOx-MOx)1 and P(iPrOx-MOx)2, respectively, c = 10 g/L). Here, similar to the reaction with NaOH, no progress of hydrolysis was observed after a few days. After 14 days, 1% of the MOx substituent in the case of P(iPrOx-MOx)1 was hydrolyzed (based on ^1^H NMR, data not shown); however, this slow reaction progress was not satisfactory. Thus, the temperature was raised to 80 °C (above T_CP_), and after 3 days, ~30% of the MOx side groups were cleaved. To further reduce the hydrolysis time, the copolymer solutions were heated at 100 °C under reflux. After 20 h at these conditions, amide bond cleavage of the MOx substituent occurred, in an amount of approximately 50% for P(iPrOx-MOx)1 and 90% in the case of P(iPrOx-MOx)2. By extending the reaction time, no further increase in the degree of hydrolysis was observed. Based on ^1^H NMR, it was confirmed that only the side groups of MOx were cleaved, maintaining the iPrOx substituent, which confirmed the selectivity of hydrolysis. ^1^H NMR spectra of exemplary P(iPrOx-MOx)2 during hydrolysis are presented in Figure 7. It is clearly seen that the signal at 1.95–2.11 ppm (protons of methyl group of MOx) decreases upon the progress of the hydrolysis. The composition of copolymers obtained after hydrolysis is shown in Table 3.

As claimed, the MOx substituents in the case of P(iPrOx-MOx)1 and P(iPrOx-MOx)2 were probably shielded by the isopropyl groups in the solution, and thus, hydrolysis at low temperature (below T_CP_), even at stricter conditions by using KOH, proceeded very slowly. An increase in temperature shortened the time of hydrolysis; thus, it can be concluded that the coil-to-globule transition in the case of thermoresponsive poly(2-oxazoline)s does not prevent the cleavage of hydrolyzable groups when a relatively strong base is applied.

### 3.3. Thermal and Crystalline Properties of iPrOx/MOx/Ethyleneimine (EI) Copolymers

**Figure 8:**
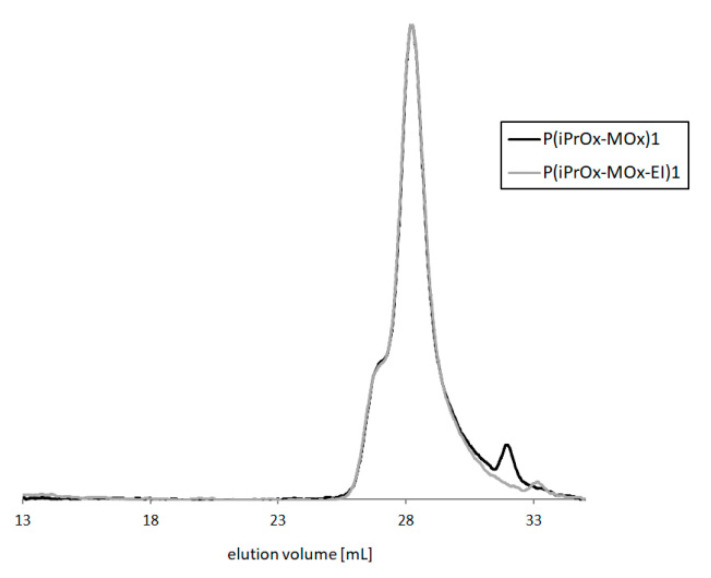
GPC traces of P(iPrOx-MOx)1 and P(iPrOx-MOx-EI)1 (DMF with 5 mmol LiBr as eluent, 1 mL/min, RI signal).

Depending on the hydrolysis conditions, copolymers of different amounts of ethyleneimine units were obtained. After this reaction, the copolymers maintained their narrow M_w_/M_n_, which means that the polymer backbone was not cleaved, and no degradation of the main chain occurred. Figure 8 presents GPC traces of exemplary copolymers before and after hydrolysis (P(iPrOx-MOx)1 and P(iPrOx-MOx-EI)1, respectively).

#### 3.3.1. iPrOx/MOx/EI Copolymers in the Solid State

**Figure 9:**
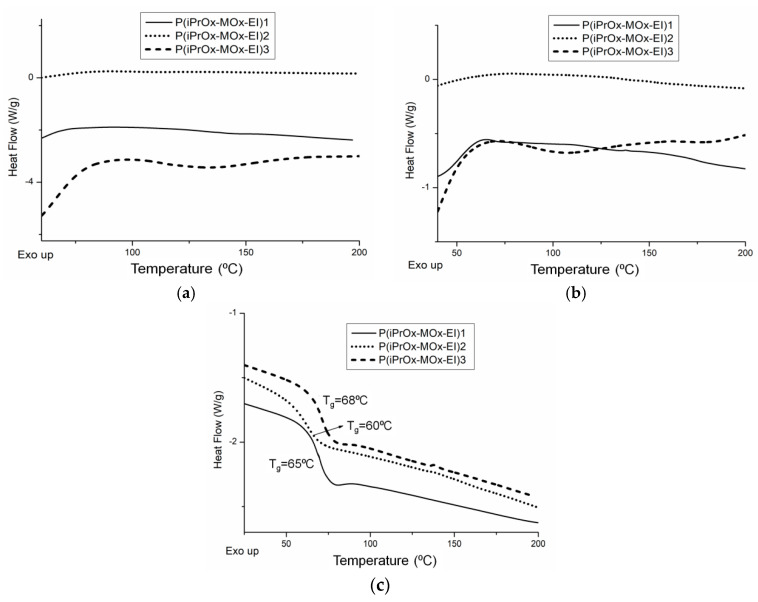
(**a**) DSC traces for iPrOx/MOx/EI copolymers at heating rates of 10 °C/min and (**b**) 2.5 °C/min; (**c**) second DSC heating run (10 °C/min) after quenching of the copolymers.

The thermal and crystalline properties of iPrOx/MOx/EI copolymers in the solid state were analyzed directly after hydrolysis (copolymers without “thermal history”) and after annealing at certain time and temperature. DSC traces for copolymers directly after hydrolysis are presented in Figure 9a. For all copolymers, neither exo- nor endothermic peaks, derived from crystallization and melting, respectively, could be seen under the standard measurement conditions (heating rate of 10 °C/min), similar to the case of copolymers before hydrolysis. At the “slow-heating” DSC measurement (heating rate of 2.5 °C/min), none of the hydrolyzed copolymers exhibited melting peaks (Figure 9b), while in the case of the copolymer P(iPrOx-MOx)1 before hydrolysis, a small endothermic peak could be seen (Figure 1b). This indicated that even a very low amount of EI units (5 mol%) lowers the ability to crystallize compared with copolymers before hydrolysis. Cleavage of the substituent in MOx units caused a noticeable decrease in the glass transition of the copolymers (Figure 9c, Table 4). This is a result of increased elasticity of the chain induced by EI segments. The most pronounced change in T_g_ of 10 °C was observed for the copolymer, where almost all substituents of MOx units were removed (P(iPrOx-MOx-EI)2).

**Figure 10:**
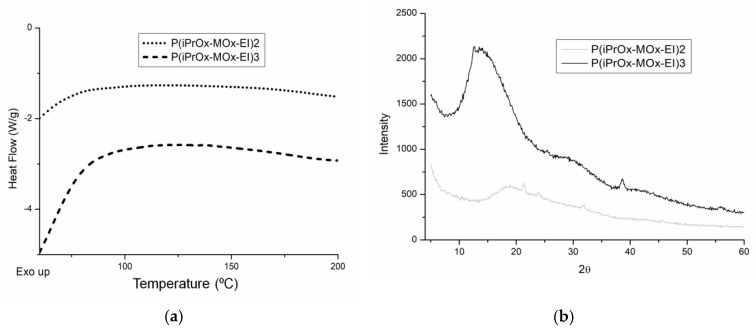
(**a**) DSC traces (heating rate of 10 °C/min) and (**b**) X-ray diffraction curves for iPrOx/MOx/EI copolymers annealed at 150 °C for 1 h.

To check whether the hydrolysis affected the crystallization ability of the copolymers, they were annealed at 150 °C for 1 h and then cooled slowly to room temperature at a rate of 10 °C/min. After this thermal treatment, a lack of melting or crystallization peaks in the DSC curve (examples in Figure 10a) was evident, as well as an absence of diffraction peaks in the XRD curve (examples in Figure 10b). Conversely, the ability to crystallize non-hydrolyzed copolymers increased drastically after similar thermal treatment (Figure 2). This confirmed that the presence of EI units caused a decrease in the crystallization ability of the copolymers in the solid state.

#### 3.3.2. iPrOx/MOx/EI Copolymers in Aqueous Solution

**Figure 11:**
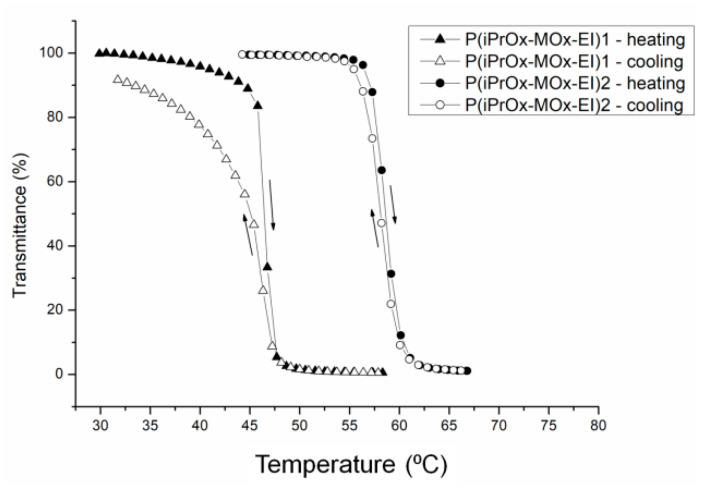
Transmittance-temperature dependence of 5 g/L aqueous solutions of copolymers after hydrolysis.

P(iPrOx-MOx)1 and P(iPrOx-MOx)2 copolymers maintained their thermoresponsiveness after hydrolysis (Table 5). The increased values of T_CP_ in copolymers subjected to hydrolysis compared to copolymers before hydrolysis are a result of the presence of EI units of higher affinity to water than MOx. The most pronounced growth in T_CP_ value was observed for copolymers where the vast majority of MOx side groups had been hydrolyzed (P(iPrOx-MOx-EI)2). The precipitation of copolymers in water was completely reversible when the solutions after coil-to-globule transition (and reaching the lowest value of transmittance) were directly cooled to room temperature. No detectable hysteresis of phase transition could be seen for iPrOx/MOx/EI copolymers, similar to before hydrolysis (Figure 11).

**Figure 12:**
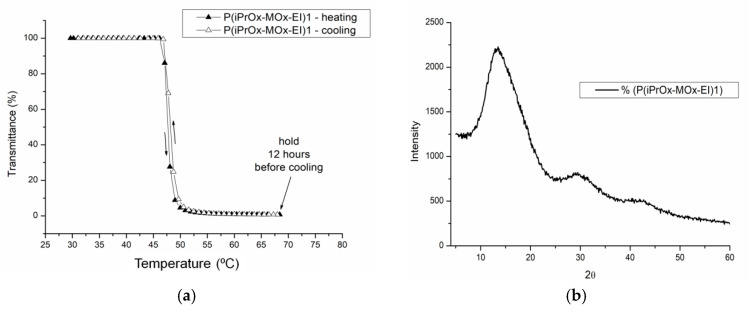
(**a**) Transmittance-temperature dependence of aqueous solutions of P(iPrOx-MOx-EI)1 (c = 5 g/L) with 12 h of incubation at 70 °C before cooling and (**b**) X-ray diffraction curves of P(iPrOx-MOx-EI)1 incubated for 12 h at 70 °C.

The aqueous solutions of P(iPrOx-MOx-EI)1 and P(iPrOx-MOx-EI)2 were then incubated in the polymer-rich phase state (at 70 °C) for 12 h. Similar to the case of copolymers before hydrolysis, a full return to transparency of the solutions was observed when the temperature was lowered after this time below T_CP_ (example in Figure 12a). Notably, no hysteresis of the phase transition was observed for hydrolyzed copolymers. The lack of crystalline phase in the solutions of iPrOx/MOx/EI was confirmed by WAXS and is presented on an exemplary P(iPrOx-MOx-EI)1 in Figure 12b.

**Figure 13:**
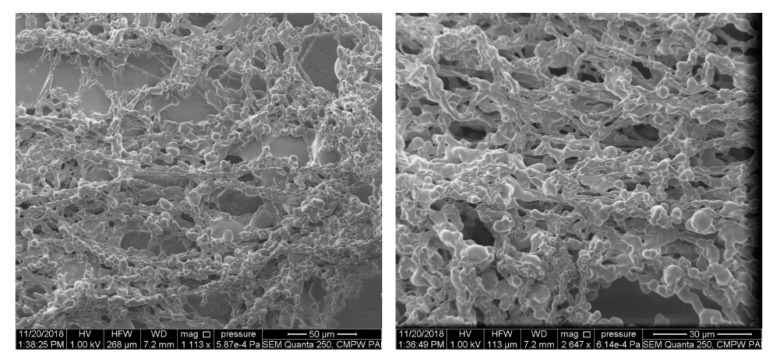
SEM micrographs of P(iPrOx-MOx-EI)2 annealed in water for 12 h at the polymer-rich phase state.

SEM analysis of iPrOx/MOx/EI copolymers incubated in water at the polymer rich-phase state for prolonged time revealed a lack of ordered structures, similar to the case of copolymers before hydrolysis (Figure 13).

Under the conditions applied in this work, we did not observe crystallization of the gradient copolymers based on iPrOx, MOx and EI both in the solid state and in aqueous solutions. Thus, it appears that these copolymers may have promising applications when compared to non-hydrolyzed iPrOx/MOx copolymers and to the iPrOx homopolymer. Additionally, the lack of hysteresis of the phase transition after prolonged incubation of hydrolyzed copolymers at the polymer rich-phase state seems to be beneficial, considering applications where the cloud point is needed at strictly defined temperatures.

### 3.4. Comparison of iPrOx/MOx/EI Copolymers with Other Polymers Used in Biological Systems

It is well known that in the case of ethyleneimine-based copolymers dissolved in different solutions at the appropriate pH, the nitrogen atom of EI units can be protonated, thus gaining a positive charge. This feature was used for the formation of polymeric complexes with nucleic acids, known as polyplexes. The principle of the formation of polyplexes is based on the presence of electrostatic interactions between the positively charged imino groups of the EI-based polymer and the negatively charged phosphate groups of nucleic acid. Among EI/poly(2-oxazoline) systems, partially hydrolyzed poly(2-n-propyl-2-oxazoline) (PnPrOx) [33], poly(2-isopropyl-2-oxazoline) [13] and copolymers of 2-methyl- and 2-phenyl-2-oxazoline [37] were applied for the formation of polyplexes. In all these systems, the aggregation of the partially hydrolyzed poly(2-oxazoline) was necessary prior to the formation of complexes with DNA. The aggregation of POx caused relocation of the positive charges derived from the nitrogen atom of EI units to the outermost surface of the structure; thus, changes in ζ-potential were observed, from close to zero/slightly negative [33] or slightly positive [13] to strongly positive values [13,33,37]. After aggregation, the positive charges derived from the nitrogen atom of EI units were easily accessible for oppositely charged nucleic acid molecules. As in many cases, an aggregation is carried out at elevated temperature; thus, it is desirable that hydrolyzed poly(2-oxazoline) does not crystallize upon incubation during reaction with DNA.

In these studies, to determine whether the iPrOx/MOx/EI copolymers obtained could potentially be used for complexation with nucleic acids, we analyzed changes in their ζ-potential and cytotoxicity.

For the aqueous solution of the hydrolyzed copolymer P(iPrOx-MOx-EI)1 at 25 °C (pH = 7.30), we observed negative zeta potential values of approximately −15 mV (±0.7). Under such conditions, EI units are deprotonated; hence, a slight negative charge could be expected. The high value of negative charge obtained is rather surprising. More importantly, at 65 °C (above T_CP_), the zeta potential of P(iPrOx-MOx-EI)1 significantly increased to a positive value of +12 mV (±0.3). This could indicate, according to the literature, that probable reorganization of the hydrolyzed copolymer in water at this temperature caused relocation of the positive charges from EI units to the outermost area of the structure formed. This issue certainly requires more detailed studies, including the organization of hydrolyzed copolymers in water at different pH levels and temperatures. However, although we did not study the aggregation behavior, because this was not the subject of this research, we can conclude that the change in ζ-potential of iPrOx/MOx/EI copolymers under the influence of temperature seems to be beneficial for the formation of polyplexes.

The toxicity of the initial polymers containing a positive charge is a very important factor when studying the efficiency of the transfection process. This was the reason for testing the toxicity of the macromolecules obtained to estimate the possibility of their use in such studies.

**Figure 14:**
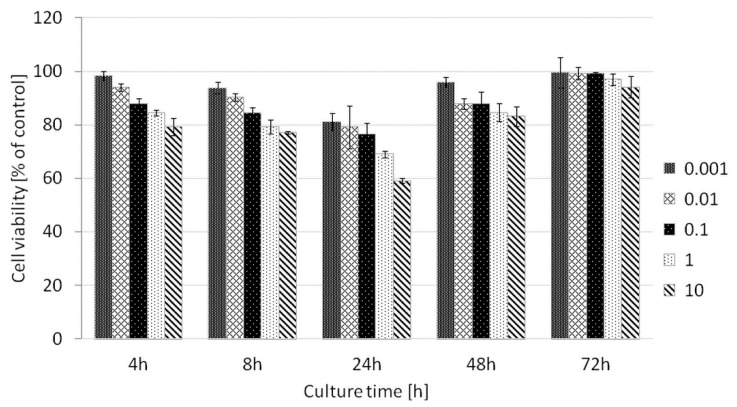
The cytotoxicity assay of the tested polymer (P(iPrOx-MOx-EI)1 at increasing concentrations (given in mg/mL). The assay was performed with HT-1080 cells. The results are shown as a percentage of the control, where untreated cells constituted 100%.

For the hydrolyzed copolymer P(iPrOx-MOx-EI)1, toxicity tests against the model cancer line HT-1080 were performed. HT-1080 cells originate from human fibrosarcoma, a tumor from fibrous connective tissue. They are often used as models in transfection studies performed with various gene delivery systems [42,43,44]. The viability of the HT-1080 cells was assessed by an Alamar Blue reduction test assay. The results are shown in Figure 14. The hydrolyzed copolymer P(iPrOx-MOx-EI)1 is non-toxic to HT-1080 cells in a wide range of concentrations. This fact opens the route to the application of this copolymer for efficient nucleic acid delivery while maintaining the lowest possible cytotoxicity of the studied system.

## 4. Conclusions

Herein, we have shown that the selective hydrolysis of 2-oxazoline copolymers strongly influences their crystallization ability both in the solid state and in aqueous solutions. For this purpose, three gradient copolymers of 2-isopropyl-2-oxazoline and 2-methyl-2-oxazoline with various compositions of comonomers were synthesized via cationic ring-opening polymerization. The subsequent selective hydrolysis, performed under basic conditions and at elevated temperature, yielded copolymers with ethyleneimine segments in the copolymer structure.

Thermal tests carried out before hydrolysis for iPrOx/MOx copolymers already showed that 2-methyl-2-oxazoline units decrease their crystallization compared to the iPrOx homopolymer; however, after sample annealing, the crystallization of these copolymers is still possible.

The hydrolysis of the obtained copolymers performed did not cause crystallization under the applied conditions, which we attribute to the increased elasticity of the chains induced by EI segments. The measurements performed after annealing experiments, both in the solid state and in water at temperatures above the phase transition, also confirmed the total suppression of this process, which proves that the hydrolysis of polyoxazolines may be an effective tool for decreasing their ability to crystallize.

Since the lack of a crystalline phase is important in terms of the bioapplications of polyoxazolines, we have shown that our polymers are nontoxic and, under the appropriate conditions, have a positive zeta potential, which altogether may be the starting point for the preparation of new polymeric vectors applicable in gene therapy.

## Figures and Tables

**Table 1:** Characterization data of the copolymers obtained.

Copolymer	iPrOx:MOx ^a^	M_n_ (g/mol) ^b^	*dn/dc* (mL/g)	*Ð*
P(iPrOx-MOx)1	90:10	10,700	0.066	1.16
P(iPrOx-MOx)2	83:17	11,700	0.068	1.16
P(iPrOx-MOx)3	49:51	9,400	0.075	1.07

^a^ Based on ^1^H nuclear magnetic resonance (NMR). ^b^ Based on GPC MALLS.

**Table 2:** T_CP_ values of copolymers at different concentrations in water.

Copolymer	c (g/L)	T_CP_ (°C)
P(iPrOx-MOx)1	10	42 ± 0.5
	5	44 ± 0.5
	1	47 ± 0.5
P(iPrOx-MOx)2	10	43 ± 0.5
	5	46 ± 0.5
	1	49 ± 0.5
P(iPrOx-MOx)3	5	>100

**Table 3:** The composition of copolymers obtained after hydrolysis.

Copolymer before Hydrolysis	iPrOx:MOx *	Hydrolysis Conditions	Copolymer after Hydrolysis	iPrOx:MOx:EI *
P(iPrOx-MOx)1	90:10	KOH, 100 °C, 20 h	P(iPrOx-MOx-EI)1	90:5:5
P(iPrOx-MOx)2	83:17	KOH, 100 °C, 20 h	P(iPrOx-MOx-EI)2	83:2:15
P(iPrOx-MOx)3	49:51	NaOH, 65 °C, 14 days	P(iPrOx-MOx-EI)3	49:39:12

* Based on ^1^H NMR.

**Table 4:** T_g_ of copolymers before and after hydrolysis.

Before Hydrolysis			After Hydrolysis		
Copolymer	iPrOx:MOx *	T_g_ (°C)	Copolymer	iPrOx:MOx:EI *	T_g_ (°C)
P(iPrOx-MOx)1	90:10	68	P(iPrOx-MOx-EI)1	90:5:5	65
P(iPrOx-MOx)2	83:17	70	P(iPrOx-MOx-EI)2	83:2:15	60
P(iPrOx-MOx)3	49:51	73	P(iPrOx-MOx-EI)3	49:39:12	68

* Based on ^1^H NMR.

**Table 5:** T_CP_ values of aqueous solutions of copolymers after hydrolysis at different concentrations.

Copolymer	c (g/L)	T_CP_ (°C)
P(iPrOx-MOx-EI)1	5	46 ± 0.5
	1	50 ± 0.5
P(iPrOx-MOx-EI)2	5	58 ± 0.5
	1	62 ± 0.5
P(iPrOx-MOx-EI)3	5	>100

## References

[1] Schlaad H., Diehl C., Gress A., Meyer M., Demirel A.L., Nur Y., Bertin A. (2010). Poly(2-oxazoline)s as smart bioinspired polymers. Macromol. Rapid Commun..

[2] Adams N., Schubert U.S. (2007). Poly(2-oxazolines) in biological and biomedical application contexts. Adv. Drug Deliv. Rev..

[3] Lorson T., Lübtow M.M., Wegener E., Haider M.S., Borova S., Nahm D., Jordan R., Sokolski-Papkov M., Kabanov A.V., Luxenhofer R. (2018). Poly(2-oxazoline)s based biomaterials: A comprehensive and critical update. Biomaterials.

[4] Luxenhofer R., Han Y., Schulz A., Tong J., He Z., Kabanov A.V., Jordan R. (2012). Poly(2-oxazoline)s as polymer therapeutics. Macromol. Rapid Commun..

[5] Hoogenboom R. (2009). Poly(2-oxazoline)s: A polymer class with numerous potential applications. Angew. Chem. Int. Ed..

[6] Huber S., Jordan R. (2008). Modulation of the lower critical solution temperature of 2-Alkyl-2-oxazoline copolymers. Colloid Polym. Sci..

[7] Park J.S., Akiyama Y., Winnik F.M., Kataoka K. (2004). Versatile synthesis of end-functionalized thermosensitive poly (2-isopropyl-2-oxazolines). Macromolecules.

[8] Gaertner F.C., Luxenhofer R., Blechert B., Jordan R., Essler M. (2007). Synthesis, biodistribution and excretion of radiolabeled poly(2-alkyl-2-oxazoline)s. J. Control Release.

[9] Kronek J., Luston J., Kronekova Z., Paulovicova E., Farkas P., Petrencıkova N., Paulovicova L., Janigova I. (2010). Synthesis and bioimmunological efficiency of poly(2-oxazolines) containing a free amino group. J. Mater. Sci. Mater. Med..

[10] Kronek J., Kronekova Z., Luston J., Paulovicova E., Paulovicova L., Mendrek B. (2011). In vitro bio-immunological and cytotoxicity studies of poly(2-oxazolines). J. Mater. Sci. Mater. Med..

[11] Bauer M., Lautenschlaeger C., Kempe K., Tauhardt L., Schubert U.S., Fischer D. (2012). Poly(2-ethyl-2-oxazoline) as Alternative for the stealth polymer poly(ethylene glycol): Comparison of in vitro cytotoxicity and hemocompatibility. Macromol. Biosci..

[12] Luxenhofer R., Sahay G., Schulz A., Alakhova D., Bronich T.K., Jordan R., Kabanov A.V. (2011). Structure-property relationship in cytotoxicity and cell uptake of poly(2-oxazoline) amphiphiles. J. Control Release.

[13] Toncheva-Moncheva N., Veleva-Kostadinova E., Tsvetanov C., Momekova D., Rangelov S. (2017). Preparation and properties of positively charged mesoglobules based on poly(2-isopropyl-2-oxazoline) and evaluation of their potential as carriers of polynucleotides. Polymer.

[14] Oleszko N., Wałach W., Utrata-Wesołek A., Kowalczuk A., Trzebicka B., Klama-Baryła A., Hoff-Lenczewska D., Kawecki M., Lesiak M., Sieroń A.L. (2015). Controlling the crystallinity of thermoresponsive poly(2-oxazoline)-Based nanolayers to cell adhesion and detachment. Biomacromolecules.

[15] Dworak A., Utrata-Wesołek A., Oleszko N., Wałach W., Trzebicka B., Anioł J., Sieroń A.L., Klama-Baryła A., Kawecki M. (2014). Poly(2-substituted-2-oxazoline) surfaces for dermal fibroblasts adhesion and detachment. J. Mater. Sci. Mater. Med..

[16] Litt M., Rahl F., Roldan L.G. (1969). Polymerization of cyclic imino ethers. VI.X-ray study of some polyaziridines. J. Polym. Sci..

[17] Sun S., Wu P. (2015). Conformational changes in the heat-induced crystallization of poly(2-isopropyl-2-oxazoline) in the solid state. Phys. Chem. Chem. Phys..

[18] Katsumoto Y., Tsuchiizu A., Qiu X., Winnik F.M. (2012). Dissecting the mechanism of the heat-induced phase separation and crystallization of poly(2-isopropyl-2-oxazoline) in water through vibrational spectroscopy and molecular orbital calculations. Macromolecules.

[19] Sun S., Wu P. (2015). From globules to crystals: A spectral study of poly(2-isopropyl-2-oxazoline) crystallization in hot water. Phys. Chem. Chem. Phys..

[20] Li T., Tang H., Wu P. (2015). Molecular evolution of poly(2-isopropyl-2-oxazoline) aqueous solution during the liquid–liquid phase separation and phase transition process. Langmuir.

[21] Özaltın T.F., Aviyente V., Atılgan C., Demirel L. (2017). Multiscale modeling of poly(2-isopropyl-2-oxazoline) chains in aqueous solution. Eur. Polym. J..

[22] Morimoto N., Obeid R., Yamane S., Winnik F.M., Akiyoshi K. (2009). Composite nanomaterials by self-assembly and controlled crystallization of poly(2-isopropyl-2-oxazoline)-grafted polysaccharides. Soft Matter.

[23] Diehl C., Cernoch P., Zenke I., Runge H., Pitschke R., Hartmann J., Tiersch B., Schlaad H. (2010). Mechanistic study of the phase separation/crystallization process of poly(2-isopropyl-2-oxazoline) in hot water. Soft Matter.

[24] Meyer M., Antonietti M., Schlaad H. (2007). Unexpected thermal characteristics of aqueous solutions of poly(2-isopropyl-2-oxazoline). Soft Matter.

[25] Diehl C., Schlaad H. (2009). Polyoxazoline-based crystalline microspheres for carbohydrate-protein recognition. Chem. Eur. J..

[26] Oleszko-Torbus N., Wałach W., Utrata-Wesołek A., Dworak A. (2017). Control of the crystalline properties of 2-isopropyl-2-oxazoline copolymers in condensed state and in solution depending on the composition. Macromolecules.

[27] Legros C., De Pauw-Gillet M.C., Tam K.C., Taton D., Lecommandoux S. (2015). Crystallisation-driven self-assembly of poly(2-isopropyl-2-oxazoline) -*block*-poly(2-methyl-2-oxazoline) above the LCST. Soft Matter.

[28] Oleszko N., Utrata-Wesołek A., Wałach W., Libera M., Hercog A., Szeluga U., Domański M., Trzebicka B., Dworak A. (2015). Crystallization of poly(2-isopropyl-2-oxazoline) in organic solutions. Macromolecules.

[29] Demirel A.L., Meyer M., Schlaad H. (2007). Formation of polyamide nanofibers by directional crystallization in aqueous solution. Angew. Chem..

[30] Lambermont-Thijs H.M.L., van der Woerdt F.S., Baumgaertel A., Bonami L., Du Prez F.E., Schubert U.S., Hoogenboom R. (2010). Linear poly(ethylene imine)s by acidic hydrolysis of poly(2-oxazoline)s: Kinetic screening, thermal properties, and temperature-induced solubility transitions. Macromolecules.

[31] De la Rosa V.R., Bauwens E., Monnery B.D., De Geest B.G., Hoogenboom R. (2014). Fast and accurate partial hydrolysis of poly(2-ethyl-2-oxazoline) into tailored linear polyethylenimine copolymers. Polym. Chem..

[32] Van Kuringen H.P.C., Lenoir J., Adriaens E., Bender J., De Geest B.G., Hoogenboom R. (2012). Partial hydrolysis of poly(2-ethyl-2-oxazoline) and potential implications for biomedical applications?. Macromol. Biosci..

[33] Mees M., Haladjova E., Momekova D., Momekov G., Shestakova P.S., Tsvetanov C.B., Hoogenboom R., Rangelov S. (2016). Partially hydrolyzed poly(*n*-propyl-2-oxazoline): Synthesis, aqueous solution properties, and preparation of gene delivery systems. Biomacromolecules.

[34] Vlassi E., Papagiannopoulos A., Pispas S. (2018). Hydrolyzed poly(2-plenyl-2-oxazoline)s in aqueous media and biological fluids. Macromol. Chem. Phys..

[35] Lambermont-Thijs H.M.L., Heuts J.P.A., Hoeppener S., Hoogenboom R., Schubert U.S. (2011). Selective partial hydrolysis of amphiphilic copoly(2-oxazoline)s as basis for temperature and pH responsive micelles. Polym. Chem..

[36] van Kuringen H.P.C., de la Rosa V.R., Fijten M.W.M., Heuts J.P.A., Hoogenboom R. (2012). Enhanced selectivity for the hydrolysis of block copoly(2-oxazoline)s in ethanol–water resulting in linear poly(ethylene imine) copolymers. Macromol. Rapid Commun..

[37] Vlassi E., Pispas S. (2015). Solution Behavior of Hydrolyzed Gradient Methyl/Phenyl Oxazoline Copolymers and Complexation with DNA. Macromol. Chem. Phys..

[38] Litt M.H., Lin C.S. (1992). Selective hydrolysis of oxazoline block copolymers. J. Polym. Sci. Part A Polym. Chem..

[39] Witte H., Seeliger W. (1974). Cyclische imidsaureester aus nitrilen und aminoalkoholen. Justus Liebigs Ann. Chem..

[40] Oleszko-Torbus N., Utrata-Wesołek A., Wałach W., Dworak A. (2017). Solution behavior of thermoresponsive random and gradient copolymers of 2-n-propyl-2-oxazoline. Eur. Polym. J..

[41] Hoogenboom R., Lambermont-Thijs H.M.L., Jochems M.J.H.C., Hoeppener S., Guerlain C., Fustin C.A., Gohy J.F., Schubert U.S. (2009). A schizophrenic gradient copolymer: Switching and reversing poly(2- oxazoline) micelles based on UCST and subtle solvent changes. Soft Matter.

[42] Hatakeyama H., Akita H., Kogure K., Oishi M., Nagasaki Y., Kihira Y., Ueno M., Kobayashi H., Kikuchi H., Harashima H. (2007). Development of a novel systemic gene delivery system for cancer therapy with a tumor-specific cleavable PEG-lipid. Gene Ther..

[43] Writer M.J., Marshall B., Pilkington-Miksa M.A., Barker S.E., Jacobsen M., Kritz A., Bell P.C., Lester D.H., Tabor A.B., Hailes H.C. (2004). Targeted gene delivery to human airway epithelial cells with synthetic vectors incorporating novel targeting peptides selected by phage display. J. Drug Target..

[44] Fus-Kujawa A., Teper P., Botor M., Klarzynska K., Sieroń Ł., Verbelen B., Smet M., Sieroń A.L., Mendrek B., Kowalczuk A. (2020). Functional star polymers as reagents for efficient nucleic acids delivery into HT-1080 cells. Int. J. Polym. Mater. Polym. Biomater..

